# Stakeholder mapping for a complex and diverse population: methodology for identifying leaders across sub-Saharan Africa

**DOI:** 10.1186/s12889-025-23026-2

**Published:** 2025-05-16

**Authors:** Katherine Banchoff, Sualiha Abdulkader Muktar, Kelly E. Perry, Kendra N. Williams, Malanto Rabary, Choolwe Jacobs, Rosemary Morgan, Anna Kalbarczyk

**Affiliations:** 1 Department of International Health, Johns Hopkins Bloomberg School of Public Health, Baltimore, MD USA; 2 International Institute for Primary Health Care Ethiopia, Addis Ababa, Ethiopia; 3 Independent Consultant, Antananarivo, Madagascar; 4 University of Zambia, Lusaka, Zambia

**Keywords:** Women’s leadership, Immunization, RMNCAH-N, Global health, Sampling, Stakeholder mapping, Sampling frame

## Abstract

**Background:**

Stakeholder-related methodologies for low- and middle-income countries (LMICs) have primarily focused on stakeholder engagement or identification of specific, well-defined populations. Current stakeholder mapping research methods do not provide sufficient sampling processes for defining and implementing a sampling frame for poorly defined populations. In this paper we develop a unique stakeholder mapping methodology and apply it to the Transforming health: The role and impact of women's leadership in the health sector (THRIVE) study, aimed at generating evidence to support investment in women’s leadership in global health decision-making in reproductive, maternal, newborn, child, and adolescent health, and nutrition (RMNCAH-N) and immunization across sub-Saharan Africa (SSA). Though current literature has examined challenges women have faced to reach leadership roles, there are no methods for systematically identifying women leaders, and leaders in RMNCAH-N and immunization have not been uniformly well-defined or systematically documented. Consequently, understanding the impact of women’s leadership on health and healthcare policies is lacking.

**Results:**

We developed a stakeholder mapping methodology to ensure accurate identification and representation of leaders in RMNCAH-N and immunization in Sub-Saharan Africa who could serve as a “sampling universe” for further investigation into the impact of women leaders. We began by defining what constituted a “leader” and “leader-adjacent” individual. Using a matrix, we refined the target sample of stakeholders and created uniform inclusion criteria. Stakeholder mapping was guided by the following strategic steps for each SSA country: screen government webpages; contact UN/multilateral agencies; conduct a systematic Google and social media search; identify relevant academic and grey literature; contact professional and personal connections in SSA; cross-check leads against a pre-defined matrix of stakeholder levels; and in-country validation. Inputs were collated into a shared Excel sheet. At the end of the stakeholder mapping exercise, we had systematically identified 3,901 leads. On average, 81 stakeholders were identified for each country. Approximately 38% (*n* = 1353) of the identified individual stakeholders were women.

**Conclusions:**

This paper’s focus on creating a sampling universe of women leaders in RMNCAH-N and immunization in SSA fills a gap in current operational and implementation research. The insights derived from the adaptation and application of this methodology highlight the value of a structured approach to capturing the complexities of stakeholder and leadership dynamics in global health, particularly when applied to systematically map health topics or disciplines that lack databases or public records.

## Background

Stakeholder mapping identifies stakeholders–people or groups with a ‘stake’ in an issue–and examines their “relative power, influence and interests” related to a specific topic, generating knowledge on how stakeholders’ interests and activities may affect or be affected by said topic [1]. Stakeholder mapping can inform research development and implementation, determine channels for participatory research through stakeholder engagement, assess potential risk or conflict areas, and monitor changes in stakeholder interests and influence over time [1]. Comprehensive stakeholder mapping efforts can also serve as the foundation for uncovering emerging issues within a discipline—particularly through prioritizing stakeholders who have not traditionally been involved at global and national levels.

Three theoretical approaches support stakeholder mapping: “descriptive accuracy, instrumental power, and normative validity.” The descriptive approach to stakeholder mapping may be applied to understand the relationship between a topic and its stakeholders while the instrumental approach values understanding how stakeholders and their behavior can influence outcomes and goals. The normative approach uses a moral argument for stakeholder involvement and empowerment, viewing stakeholder mapping as a tool to identify, uplift, and provide a platform for marginalized stakeholders to influence decisions made by those who hold more power [2, 3, 4, 5, 6] These theoretical approaches can be extended and applied to research methodologies, given that research influences decision-making and informs policy, practice, and further research.

Stakeholder mapping methodologies have traditionally been employed in management, policy, and educational disciplines in high-income countries (Preston and Sapienza 1990; Brugha and Varvasovszky 2000). Stakeholder-related methodologies for low- and middle-income countries (LMICs) have primarily discussed stakeholder engagement and involvement rather than identification (LMG Project Team 2014) or have focused on identifying specific, well-defined populations, such as men who have sex with men and undocumented immigrants (Kendall et al. 2008; Reichel and Morales 2017). While aspects of these approaches may be adapted for global health initiatives, current research methods do not provide sufficient sampling processes for defining and implementing a sampling frame for participants across diverse geographies, organizations, and professional statuses in biomedical and social sciences [7, 8], presenting a barrier to sampling those who do not fit neatly into traditional categories or well-defined groups. Stakeholder identification in high-income countries (HICs) often benefits from the existence of structured databases, professional directories, and formal records. In LMICs, such comprehensive datasets are not always available or complete. Similarly, HIC methodologies may use quantitative metrics to evaluate stakeholders’ power, influence, or salience whereas such metrics are less applicable or adept at capturing nuances in leadership in LMIC contexts. These methods struggle to capture populations which lack uniformity and cannot be identified by standardized classifications or heterogeneous characteristics. When target and source populations lack specificity and are poorly defined, as is often the case within complex health initiatives, data may be extrapolated based on sampling frames that lack representativeness and lessons learned may be missed given the lack of granularity at various leadership and organizational levels.

This gap is illustrated by the Transforming health: The role and impact of women's leadership in the health sector (THRIVE) study and our experience identifying stakeholders to participate as the sampling population in our multi-country study exploring the impact of women leaders. Leaders in global health are not a well-defined population and as such, systematic mapping of leaders in global health has not yet been conducted, thus presenting a barrier to identifying stakeholders and evidencing a methodological gap. This barrier is even more pronounced in LMICs, where data– such as professional directories– may be less available than in HIC (World Bank). Commonly used stakeholder mapping frameworks also fell short of meeting the needs of the study. Stakeholder mapping using a grid with two axes, for example, Mendelow’s “power versus interest” matrix [9], can be used to quickly categorize stakeholder groups and/or individuals, facilitating identifying the most powerful or influential stakeholders (Reed and Curzon 2015). However, in the context of women’s leadership in SSA, reducing stakeholder dynamics to just two dimensions overlooks nuances of leadership. Focusing on high-power/high-interest stakeholders has the potential to neglecting groups with low power but high stakes in the outcome, such as women leaders. The salience model [10] classifies the significance of stakeholders to decision-makers based on three attributes: power, urgency, and legitimacy, similarly deprioritizing populations with legitimacy but low urgency or power.

Consequently, a tailored stakeholder mapping methodology was developed and implemented. We aim to systematically identify and sample stakeholders as a first step in the study, valuing generating knowledge on women’s leadership with and from women leaders themselves. We take an adapted normative and instrumental approach to stakeholder mapping: the study intends to detail the impact women leaders’ have on specific outcomes and amplify their voices, as the study may affect decision-making related to women’s leadership. This includes increased investment in leadership programs and initiatives for women. Though some components of well-known stakeholder mapping frameworks have guided the development of this methodology, adapting it to be more iterative, participative, and considerate of cultural influences and gendered power dynamics within leadership in SSA has been a critical approach to ensuring the methodology’s relevance in LMIC settings.

### Introduction to the women’s leadership study and stakeholder mapping

While women represent the majority of workers in the health sector globally, this is not reflected in roles of leadership in global health. Women comprise 70% of the healthcare workforce, and 90% of the nursing and midwifery workforce but they hold only 25% of leadership roles. As of 2020, only 44 women worldwide were serving as Ministers of Health [11]. A dearth of research exists on the impact of women’s leadership on health and healthcare policies and the environments that foster impactful leadership from women [12]. The generation of such evidence will support investment in women’s leadership and women leaders themselves, which will help to increase the overall representation of women leaders.

Funded by the Global Financing Facility for Women, Children and Adolescents (GFF) and Gavi, the Vaccine Alliance, the THRIVE study investigates and documents (1) the impact of women leaders on health programs and policies, decision-making/prioritization, and organizational change, particularly in reproductive, maternal, newborn, child, and adolescent health, and nutrition (RMNCAH-N) and immunization across SSA, and (2) ways to enhance the role of women leaders and promote positive transformations in the RMNCAH-N and immunization landscapes. These two fields represent key strategic focal areas of GFF and Gavi.

The study began with identifying stakeholders: both women and men leaders, “leader-adjacent” individuals, and relevant organizations and institutions in RMNCAH-N and immunization in SSA. The identified stakeholders were contacted to participate in the primary data collection activities of the larger study, elucidating the impact of women leaders, particularly on priority setting, funding and how it is distributed, health outcomes, and policy, and in particular how this is similar to or different from the impact of men leaders. An online survey was administered to all stakeholders assessing perceptions of the impact of their own leadership (if they are a woman) and/or women’s leadership in general. The research team will involve women leaders by conducting qualitative semi-structured interviews exploring these impact areas in more depth. As it is challenging to quantify the impact of women leaders across the entirety of Sub-Saharan Africa, the THRIVE study focuses on leaders’ own reports or perceptions of either their personal impact or the impact of women’s leadership more broadly. A sub-group of identified stakeholders were engaged for in-country validation of the sample. All stakeholders will be invited to share recruitment materials among their networks, contributing to snowball sampling. The insights gleaned from stakeholders’ involvement as research participants and supporters were used to inform the study’s findings and subsequently guide broader investment, policy, and programming geared at women leaders in RMNCAH-N and immunization. Furthermore, stakeholders will be invited to leverage their networks and collaborate in disseminating the study’s findings. They were mobilized to advocate for the implementation of study findings within their workplaces and at the local, national, and regional levels. By engaging stakeholders in advocacy and dissemination, the study aims to share ownership of the findings with the people involved and nourish a Sub-Sahara African network of advocates for gender equity and leadership in RMNCAH-N and immunization.

Stakeholder mapping within the context of this study: (1) provided a sample of individuals involved in RMNCAH-N and immunization across levels of leadership, and (2) served as an overview of the landscape of the potential leaders in RMNCAH-N and immunization. To identify potential study participants, we conducted an extensive stakeholder mapping exercise, described below.

## Methodology

The research team consisted of four researchers with English, French, Portuguese, and Amharic language abilities as well as diverse geographic expertise in SSA (based on the World Bank definition). The team conducted stakeholder mapping over two months (January-February 2024). Each team member was assigned between 6 and 17 countries. French- and Portuguese-speaking team members led the stakeholder mapping process for Francophone and Lusophone countries.

### Defining our sample

As we did not have access to a pre-existing database on our targeted sample (which other stakeholder mapping methods typically rely on), such as comprehensive databases or public records (Kalleberg et al. 1990), we developed a unique stakeholder mapping methodology. This methodology allowed us to ensure accurate identification and representation of leaders in RMNCAH-N and immunization. To systematically identify our sample of stakeholders, we began by clearly defining who could be considered a “leader” (Box 1) as well as “leader-adjacent” individuals who may not meet our definition for “leader” but may be in contact with them and could serve as key entry points to connecting with leaders for follow-up research.

**Box 1:** Leadership definition

Leaders in immunization and RMNCAH-N were defined as: women or men who occupy a position which gives them influence and power over identifying priorities, providing strategic direction, allocating resources, and decision-making within the immunization and/or RMNCAH-N sector at either the sub-regional, regional, national, or continental level. To identify as women or men leaders, participants will need to answer in the affirmative to one or more of the following questions. In your current role, do you have influence over: • How decisions are made? • Which priorities are identified? • How funding is distributed? • Strategic direction of your organization or institution?	

### Framework and identification of leaders through stakeholder mapping

We identified relevant stakeholder categories by designing a matrix of state and non-state actors by organizational and operational levels (Table 1) to further pre-define the target audience and adopt a multi-source approach to identifying stakeholders in line with our leadership definition, ensuring the inclusion of diverse and representative stakeholders while maintaining focus on leadership roles. The matrix serves to avoid any ambiguity in the inclusion criteria of the sample and ensure no sub-groups of leaders are excluded. It also outlines a uniform search strategy within the team of multiple researchers conducting the mapping exercise. In-country validation and snowball sampling aimed to improve coverage and validate stakeholder data for accuracy. The study team conducted periodic reviews of the shared data sheet to address under- or overrepresentation and documented all decision-making steps to ensure reproducibility.

**Table 1:** State and Non-State actors by organizational and operational levels

Actor Type	Target Audience	Useful Documents for Snowballing
State	Head of State Ministries (E.g., Ministry of Health) Regional Health Bureaus Public Health Associations (e.g., Medical Associations, OB-GYN societies, midwifery and nursing associations, Health Officer Associations) Country EPI Country NITAGs Inter-agency coordination committees (ICCs)	Policy documents Reports News briefs Partnership lists: Development partners, Donors NGO lists (I.e. funded by government, registered with government)
Non-State: NGOs (regional/national/sub-regional/international)	Executive Board/Top Management Team Country Director/Representative, Deputy Director, Area Manager Regional Offices	Policy documents Reports News briefs Partnership/donor/funder lists
Non-State: UN/Multilateral Agencies *I.e. UN Women*, *UNICEF*, *UNHCR*, *GFF*, *WHO*, *GAVI*, *ILO*	Executive Board Regional/National Offices	Reports News briefs Partnership lists
Non-State: Academia	Research Institutions Research Working Groups Universities (e.g., United Nations University) Educational Programs	Relevant Research Papers (who funded them, what are their affiliations) Development of technical standards and guidelines Conferences (and conference abstracts): Keynote speakers, panelists, presenters, etc.
Non-State: Private Sector	Banks/financial institutions (Donors) Professional Development/ Leadership Programs	Awards ceremonies Scholarships

Recognizing that health funding and programming are frequently influenced by political dynamics, corruption, and shifts in governance, we included both state and non-state actors (NSAs) in our target audience. The World Health Organization defines non-state actors in health as nongovernmental organizations, private sector entities, philanthropic foundations and academic institutions. We employed a modified approach and replaced philanthropic foundations with UN/multilateral agencies in our matrix. Different categories of stakeholders contribute uniquely to RMNCAH-N and immunization leadership. State actors set the national health agenda, align priorities with global frameworks, strengthen the health workforce, and allocate health budget is allocated towards RMNCAH-N and immunization. Federal laws and regulations impact service delivery, including the vaccine cold chain and access to contraceptives. NSAs complement and supplement state actors by offering essential services, fillings gaps and meeting demands. This may particularly be the case for marginalized groups, regarding politically “controversial” topics, or when state actors otherwise fall short. For example, NGOs work with governments as implementation partners and advocates for RMNCAH-N and immunization inclusion in national health policies and UN/multilateral agencies work with state and non-state actors to scale programs and push for common goals across the global health agenda. Private sector entities drive innovation and offer leadership training, as well as impact service delivery and supply chains. Finally, academic institutions generate evidence to shape and inform health policy and practice, provide technical support, and advise other non-state and state actors. Leadership in each of these categories influences priorities, strategic direction, resource allocation, and decision-making. For inclusion criteria to be met, stakeholders must fit into at least one of the state or non-state categories in the matrix and fit the study’s leadership definition or be “leader-adjacent.”

**Fig. 1:**
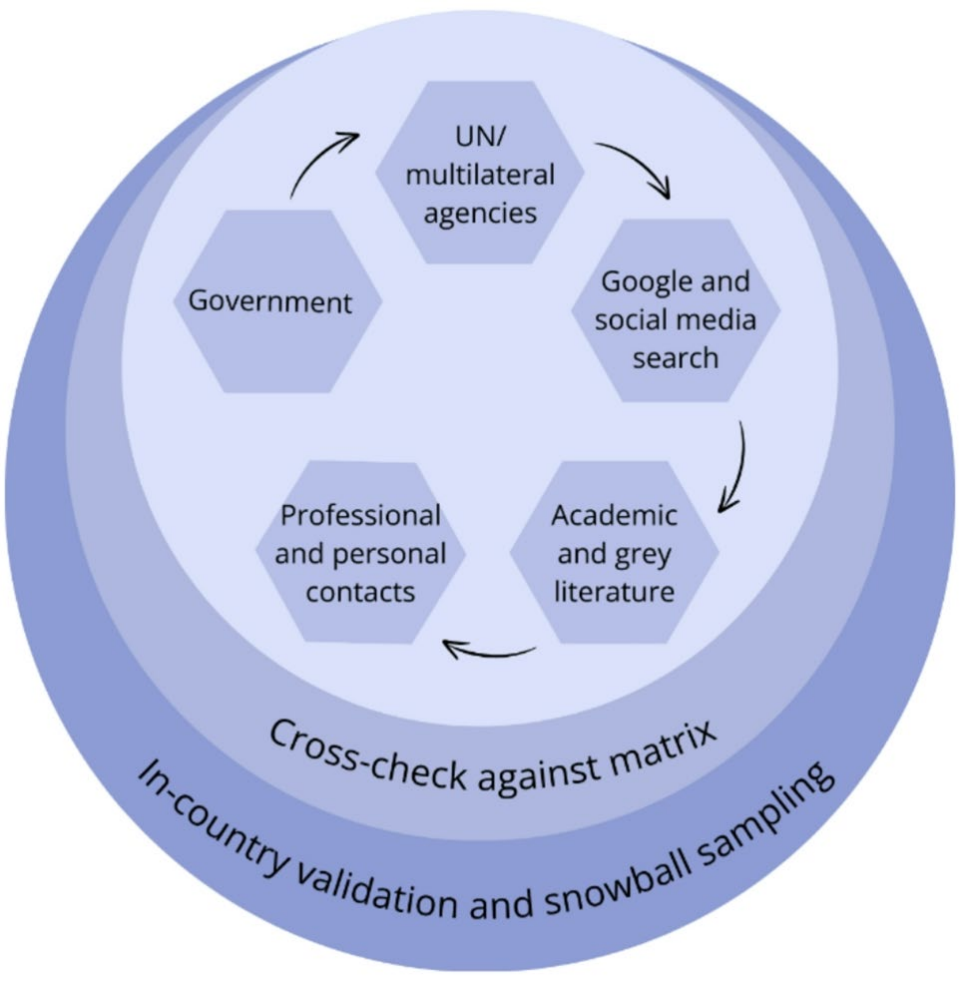
Overview of the stakeholder mapping process conducted within each Sub-Saharan African Country

After defining our sample, stakeholder mapping was guided by the following strategic steps for each SSA country: screen government webpages; contact UN/multilateral agencies; conduct a systematic Google and social media search; identify relevant academic and grey literature; contact professional and personal connections in SSA; cross-check leads; and in-country validation (Fig. 1). Inputs were collated into a shared Excel sheet. Each strategic step is detailed below.

Throughout the stakeholder mapping exercise, we held weekly calls to refine the search protocol and ensure all team members were using the same methodologies for systematically searching stakeholders. As mapping progressed, weekly calls focused on sharing mapping strategies and results, lessons learned, and identified public databases with relevant stakeholders and their contact information for each SSA country. Some stakeholders’ work in RMNCAH-N and immunization spanned multiple SSA countries and were documented accordingly.

We recorded all identified stakeholders in a shared, live Excel mapping template. The Excel template included primary contact information and employment information as well as categorizations for RMNCAH-N and immunization-relevant stakeholders. Stakeholders’ genders were documented, where available, to monitor and ensure our representation goals of at least 25–50% women leaders, in line with the current proportion of 25% women in leadership roles in global health. A quota of at least 30 stakeholders per country was set to ensure diversity of stakeholders across all SSA regions, making the study more representative of the entire SSA region rather than a single geographic sub-region. Given that our target population is poorly-documented and relatively small compared to the total population, non-probability sampling was a necessary methodological decision. This approached enabled the research team to systematically and purposively identify individuals with the study’s desired characteristics within each country, while operating within human resource and time limitations. Further demographic information, such as ethnicity and socioeconomic status, was not monitored due to being seen as irrelevant to the study goals if the stakeholders met our leadership definition. Further, we deemed it inappropriate to ask about in our primary data collection activities with leaders. Stakeholder information was arranged across four Excel sheets depending on the organizational and operational level of the contact: (1) state actors, (2) non-state actors, (3) individuals, and (4) professional and personal contacts (Table 2).

**Table 2:** Stakeholders per organizational/ operational level

Organizational/ Operational Level	Stakeholders Included
State Actors	Ministry of Health and other government entities’ websites and general contact information, including their social media information
Non-State Actors	Organizational entities and general contact information, including their social media information
Individuals	Individuals affiliated with state or non-state entities and their contact information (e.g., individual email addresses) and gender (if available)
Professional and Personal Contacts	Professional and personal connections and leads, as well as their contact information (e.g., individual email addresses) and gender (if available)

An additional two sheets were included in the Excel matrix to record notes and administrative duties for the research team. Each team member regularly populated the “Notes” sheet to document relevant search notes, challenges, and lessons learned, including “pulse checks” on using predefined strategic steps and whether any course correcting needed to occur. We recorded administrative duties and assigned responsibilities per member and per SSA country within a separate sheet.

### Screening government webpages

Each SSA country’s government webpages were screened first for the relevant RMNCAH-N and immunization departments, teams, units, and initiatives, including the Ministry of Health, Department of Women’s and Children’s Affairs, Department of Family Health, and Ministry of Social Welfare, among others. We intentionally searched for Expanded Programs on Immunization (EPI), National Immunization Technical Advisory Groups (NITAGs), and inter-agency coordination committees (ICCs) for immunization stakeholders in each country. Relevant primary contact information was collated, initially targeting the general email address for relevant departments as well as listed officials and heads of departments, where available. We also screened for listed implementation partners or otherwise government-endorsed organizations (e.g., local nonprofit organizations), including organizations that receive government funding, and any third-party donors–typically other foreign governments or multilateral agencies—within the government webpages where available. Throughout the screening, we organized country data in the Excel matrix, adding individual names and relevant contact information in addition to departmental contact information.

### Contacting UN/multilateral agencies’ country offices

We pre-identified relevant UN and multilateral agencies, including WHO, GAVI, the Global Financing Facility (GFF) within the World Bank, UNICEF, and USAID, among others. Relevant organizations were broadly defined to include those involved with funding, researching, designing, managing, and implementing activities related to RMNCAH-N and/or immunization. We then contacted their country offices and/or focal points, including Liaison Officers, requesting contacts for government stakeholders, agencies’ in-country partners and implementing organizations, and other relevant individual contacts and/or organizations. Our relationship with UN/multilateral agencies’ in-country focal points extended throughout the stakeholder mapping process as a method to periodically identify missing contact information, as well as utilize the focal points’ connections and resources to advance snowball sampling.

### Conducting systematic Google search and social media

Once relevant contacts and organizations had been identified through the government webpages and UN/multilateral agency recommendations and endorsements, we conducted a systematic Google search for additional in-country RMNCAH-N and immunization-related stakeholders. This search served to address any gaps for relevant local and international non-profit organizations and NGOs, universities, and in-country private sector organizations. In addition to compiling organizational or institutional contact information, “Leadership” or “People” pages were targeted to identify potentially relevant individuals, including within Top Management Teams, Board of Directors, and Advisory Committees webpages. We then visited social media platforms, such as LinkedIn and the “People Also Viewed” field within LinkedIn, for other potentially relevant individuals in the RMNCAH-N and immunization sectors. Contact information was continuously added to the Excel matrix.

### Identifying academic and grey literature

Expanding into academic and grey literature, we searched for publications, blog posts, and/or articles written about RMNCAH-N and/or immunization in each SSA country, identifying in-country co-authors or mentioned relevant stakeholders, and collating their contact information where available. Publications were identified via Google Scholar and relevant databases such as PubMed. If co-authors had a public social media presence, we also collated information accordingly. Again, LinkedIn pages and the “People Also Viewed” field on LinkedIn was used to identify other potentially relevant individuals and network members.

### Contacting professional and personal connections

Our research team members reached out to their professional and personal connections in SSA to fill in any gaps and provide further recommendations of individuals, organizations, and units. Professional contacts included Women in Global Health, consultants based in Ethiopia, Madagascar, and Zambia, and GFF and GAVI liaisons. The UN/multilateral agency focal points continued to support the stakeholder mapping process by cross-checking data where possible. Personal contacts included university alumni groups, past employers or coworkers, community groups, and colleagues.

### Maintenance and cross-checking

Once leaders were identified using the steps described above, they were categorized in the Excel sheet based on their involvement as, or with, potential leaders in RMNCAH-N and immunization in SSA. Categories consisted of sector, institution type, and job title. At this stage of the stakeholder mapping process, government webpages, UN/multilateral agencies, the non-profit and private sector, and academia had been systematically screened for stakeholders in RMNCAH-N and immunization in SSA. Our team’s personal and professional connections supported stakeholder identification and filled any gaps. The stakeholder mapping matrix (Table 1) was referred to continuously throughout the stakeholder mapping process to guide the team and at its conclusion, to cross-check contacts and organizations identified in each country against the inclusion criteria. Recognizing the potential for sampling bias, including selection bias and biases introduced depending on who initiated recruitment efforts (i.e. trusted and influential in-country partners as compared to the unknown study team members), the study team aimed to mitigate a potential lack of diversity in the sample by adhering to quotas for gender and geographic representation. At each team meeting, the sampling pool was assessed for any shortcomings in representativeness.

### In-country validation

At the conclusion of the stakeholder mapping exercise, he stakeholder mapping results were then sent to the GFF Liaison Officers (LOs) or in-country focal points (FPs) for in-country validation. The LOs and FPs assisted the research team in filling in any gaps, providing missing contact information, as well as identifying more potential stakeholders who could be eligible for recruitment. Colleagues already integrated into GFF teams helped us sensitize GFF LOs/FPs and share mapping results with them for input per country. Virtual meetings between our research team members and LOs/FPs helped to clarify study goals, eligibility criteria for targeted stakeholders, and orient LOs/FPs to the study. This sub-set of stakeholders were engaged for snowball sampling among their networks.

To continuously expand the sampling universe and obtain as many relevant stakeholders as possible, all stakeholders identified in the mapping process were contacted for recruitment to the larger study and asked to recommend others as part of snowball sampling for the study. Stakeholders were offered the option to forward our recruitment email to their contacts rather than sharing their contact information directly with the study team.

## Results

At the end of the stakeholder mapping period, we had systematically identified 3,901 leads. The study team set a quota of a minimum of 30 stakeholders per each of the 49 Sub-Saharan Africa countries. Ultimately, a range of 34 to 99 stakeholders were identified per country with two notable outliers: Democratic Republic of Congo (DRC) (*n* = 1144) and Ethiopia (199). A large proportion of the DRC stakeholders were identified via a public database. The study team includes an Ethiopian researcher who is based in Ethiopia and was able to leverage her network of contacts. On average, 81 stakeholders were identified for each country.

In line with our goals to identify at least 25% women leaders, 38% (*n* = 1353) of the identified individual stakeholders were women, 61% (*n* = 2148) were men, and 1% (*n* = 44) did not have gender specified. Stakeholders’ gender was inferred based on the individual’s name, photograph, and the presence of gender pronouns or honorifics such as Mr. or Ms. associated with their title. However, we recognize there is a margin of error here as not all individuals’ gender is necessarily in accordance with their appearance or name. Occasionally, we had the contact information of a team’s contact person, but not a name or gender. In some cases, the names of relevant stakeholders were identified, but their contact information was more challenging to source. Of the contact information identified, when the stakeholder list was de-deduplicated, the majority were email addresses (*n* = 2602, 72%), followed by LinkedIn profiles (*n* = 856, 24%), and telephone numbers (*n* = 142, 4%). Contact information was unavailable for 8% (*n* = 272) of stakeholders. This means that the stakeholders name appeared in at least one of the phases of the mapping activity, but ultimately an email address, phone number, or LinkedIn profile was unable to be determined. This may be because the person was a high-level leader with communications directed to assistants, they were one of many key figures credited or mentioned in a single source document, or because the person’s organizational webpage does not publish employee contact information. LinkedIn’s popularity and use may vary by factors such as age or region.

Contact information for in-country offices and staff members of UN/multilateral agencies was most easily available, as well as their in-country implementing partners. Not all SSA countries had a Ministry of Health webpage with up-to-date information. Thus, for many countries, the government’s social media presence, particularly via Facebook or X (formerly Twitter), provided the most comprehensive information on RMNCAH-N and immunization departments, initiatives, and key stakeholders, particularly via sharing updates or news articles with followers.

## Discussion

Our stakeholder mapping exercise aimed to address the gap in methods for defining and implementing a sampling process for participants across multiple global and professional contexts in biomedical and social sciences [7, 8] created a sample of potential leaders and “leader-adjacent” individuals and relevant organizational and institutional bodies in the RMNCAH-N and immunization spaces across sub-Saharan Africa. The contacts identified comprehensively represent the RMNCAH-N and immunization space in SSA and will form the basis for the recruitment of participants for the next phases of the THRIVE study.

The lack of accurate data on women leaders in global health, particularly within SSA, represents a barrier to their leadership by limiting the ability to advocate for and implement data-driven interventions and approaches. It also highlights a broader issue of women leaders’ lack of recognition in global health [13, 14]. This stakeholder mapping exercise enabled us to comprehensively sample this population, offering an opportunity to amplify women leaders’ voices in a male leadership-dominated discipline, despite the workforce being primarily female [15].

Amplifying stakeholders’ voices provides a platform for them to influence the data that affects them when it is used to inform programming, policy, and future research. By providing this platform, stakeholder mapping can challenge traditional power dynamics and move those affected to the forefront, promoting a standard of practice that applies a decolonial and feminist lens to research methodologies. The development, streamlining, and standardization of stakeholder mapping methodologies that reinforce the capacity of researchers to sample diverse or poorly defined stakeholder groups, namely those who typically hold less power in decision-making processes, can promote more research on specific, understudied populations. This not only upholds its ethical validity, but also adds value to stakeholder mapping as a functional tool (Box 2).

**Box 2:** Stakeholder mapping to advance women’s leadership: womenlift health case study

WomenLift Health commissioned a series of stakeholder analyses in North America, Europe, India, Nigeria, and East Africa (WomenLift Health 2023). To develop understanding on the unique context of each geography, they partnered with local research organizations to identify and interview key health sector stakeholders and organizations. The stakeholder analysis began by identifying the most influential organizations in public health within each country. Research teams then prioritized organizations and individual senior- or executive-level leaders within these organizations. Most respondents identified institutional-level reforms as the greatest opportunity to increase women’s leadership. Many others called for an expansion of policies or other support mechanisms for working parents. Across all focal countries, respondents highlighted the continuing need for talent development programs. The WomenLift Health stakeholder analysis exercise also aimed to promote collaborative implementation of programs to reduce the gender disparity in health sector leadership. Participants were reportedly enthusiastic about sharing the organization’s Leadership Journey opportunity with their networks and nominating potential fellows. They also expressed interest in receiving trainings from WomenLift and collaboratively facilitating or co-creating such trainings. Other stakeholders identified advocacy opportunities at both national or sector-wide levels to push for reforms that could benefit women’s representation in global health leadership.	

While methods have been developed to apply systems approaches to global health, they do not yet sufficiently guide sampling from a diverse group of stakeholders across geographies, leadership levels, and cultural contexts [16]. We sought to complement other novel approaches aimed at developing a sampling frame for a target population, particularly in LMICs or amongst undefined or poorly defined populations. Lacking data on the distribution of complex source populations in LMICs impairs planning and priority setting [17]. As such, the ability to generate this data can have policy and programming implications for marginalized and intersectional groups. Our source population mapping methodology was guided by a similar approach outlined by Peters et al. in the Global Polio Eradication Initiative (GPEI) [8], which emphasizes theorizing a target population or “universe” of actors in the study area and enumerating a source population of specific individuals within the target population. The THRIVE study adopting this approach to defining our leadership “universe” and source population across multiple organizational and operational levels of state and non-state actors (Table 3). We also incorporated contacting point persons for contact information of potential stakeholders, a strategy utilized by GPEI country teams. However, while Peters et al. emphasized estimating the size of the sampling universe, taking into consideration the operational feasibility of the sample, this study bypassed this step to inform data collection activities.

**Table 3:** A guiding framework for strategizing the stakeholder mapping sampling universe, as adapted from and inspired by Peters et al. 2020

Describe the program of interest	Define goals to map and synthesize tacit knowledge, ideas, approaches, and experiences that are not documented but are relevant to understanding the impact of women’s leadership in RMNCAH-*N* and immunization within various contexts		
Define a sampling universe to meet criteria	Organizational and operational levels (Table 1) Relevant organizations are broadly defined to include those involved with funding, researching, designing, managing, and implementing activities related to RMNCAH-N and/or immunization.	The RMCAH-N and immunization universe across the study areas was “the population of individuals who have influence and power over identifying priorities, providing strategic direction, allocating resources, and decision-making within the immunization and/or RMNCAH-N sector at either the sub-regional, regional, national, or continental level *for 12 or more continuous months between 2000 and the present.*”	
Estimate sampling universe size	(i) conservatively estimate the number of all individuals who could have possibly been involved (ii) estimate the Top Management Team (i.e. our leadership definition) workforce of key organizations identified	(i) purposively or randomly selecting geographical sub-units within SSA or within a country for enumeration (ii) utilizing snowball sampling; using existing networks to distribute the survey (iii) convening stakeholders and enumerating as many individuals as possible within key organizations	
Enumerate a source population within the universe that can be feasibly reached for sampling	Strategy involves systematically identifying relevant organizations (see definition above) across SSA through a variety of sources.	Strategy involves contacting point persons at identified key organizations and requesting contact information for individuals and organizations who may belong to the RMNCAH-N and immunization universe across SSA.	Once universes were defined and described, we use *different criteria and assumptions* for estimating the sample size taking into consideration the operational feasibility of the sample.
Sample from the source population	Administer survey to a sample of the enumerated source population.	Utilize various methods of administering the same survey tool to ensure that responses were characteristic of the enumerated source populations both in terms of quantity of responses as well as a variety of respondents.	Survey respondents and response rates can be compared with original estimates of the source population and universe to assess the comprehensiveness of the survey.
Reflect on the process to determine strength of inferences drawn	Variability in the process of operationalizing the RMNCAH-N and immunization universe, defining a source population, and data collection limits the strict generalizability of the findings of specific country surveys but yields important conclusions for the global RMNCAH-N and immunization efforts more broadly.		

The insights derived from the adaptation and application of this methodology highlight the value of a structured approach to capturing the complexities of stakeholder and leadership dynamics in global health. Our systematic mapping ensured that perspectives are collated from stakeholders with a variety of backgrounds and experiences at community, sub-national, and national levels, enabling a comprehensive approach to synthesizing lessons learned regarding the impact of women’s leadership in RMNCAH-N and immunization. In future research, this methodology could be applied to systematically map other types of stakeholder groups, with a particular focus on LMICs, health topics or disciplines that lack systematic databases or public records, and marginalized, intersectional, and disenfranchised populations. In the case of the THRIVE study’s mapping efforts, we know that research on women’s leadership is moving beyond the documentation of barriers towards advancing data-driven approaches to overcome them. Work in this field is also shifting towards creating new paradigms on how women’s leadership is enabled by women themselves, and within workplaces, communities, and societies locally and globally. While individual factors are enablers of advancing women to leadership positions, they alone are not sufficient [18]. Environmental, institutional, and individual interventions can be effective in closing the gender gap in female global health leadership [19]; however, it is from creating a comprehensive stakeholder map and effectively sampling women that we are able to highlight these solutions and ultimately mainstream them.

The stakeholder mapping exercise supports broader global health goals, namely Sustainable Development Goal (SDG) 5: Gender Equality, specifically SDG 5.5: women’s full participation and equal opportunities in leadership [20]. By systematically identifying women leaders in RMNCAH-N and immunization, this work directly supports efforts to generate evidence on the impact of women’s leadership, recognize their value, and ultimately, translate knowledge acquired through sampling processes into increased investment and programming toward gender transformative leadership and more equitable health systems. The insights generated from the ability to sample this population of leaders also contribute to SDG 3: Good Health and Well-Being [20]. This methodology contributes to improved health systems by facilitating the identification of the unique influence-to-impact pathways that enable women leaders to affect RMNCAH-N and immunization health outcomes. Understanding where and how women leaders make the most impact enables efficient allocation of resources towards initiatives that strengthen their capacity to do so.

This work contributes to broader systemic changes in global health leadership dynamics by ensuring that women in the RMNCAH-N and immunization sectors in SSA are heard and recognized for their leadership. Creating new paradigms around how women’s leadership is conceptualized in the workplace and the global health sector more broadly requires prioritizing activities that support it. Allocating resources and attention towards women’s leadership challenges existing gender inequities and barriers that persevere in global health sector. By creating a scalable framework, this research streamlines stakeholder mapping of similar populations in other sectors and regions, thus promoting inclusivity and equity in leadership dynamics more broadly. This methodology contributes to improved health systems by facilitating the identification of the unique influence-to-impact pathways that enable women leaders to affect RMNCAH-N and immunization health outcomes. Understanding where and how women leaders make the most impact enables efficient allocation of resources towards initiatives that strengthen their capacity to do so. Finally, this work contributes to broader systemic changes in global health leadership dynamics by ensuring that women in the RMNCAH-N and immunization sectors in SSA are heard and recognized for their leadership. Creating new paradigms around how women’s leadership is conceptualized in the workplace and the global health sector more broadly requires prioritizing activities that support it. Allocating resources and attention towards women’s leadership challenges existing gender inequities and barriers that persevere in global health sector. By creating a scalable framework, this research streamlines stakeholder mapping of similar populations in other sectors and regions, thus promoting inclusivity and equity in leadership dynamics more broadly.

We acknowledge some limitations to this stakeholder mapping methodology that may affect the studies reliability, validity, and applicability. Relevant contacts may have been missed by restricting the search to online documents, websites, and literature only and excluding text written in languages other than English, French, and Portuguese. The exclusion of non-colonial languages could systematically marginalize populations that primarily communicate in Niger-Congo, Afroasiatic, Nilo-Saharan, Khoisan, Austronesian languages or contact varieties, resulting in skewed dataset and findings that are not representative of the entirety of Sub-Saharan Africa’s diverse population of leaders. Findings that disproportionately reflect the views of certain language groups may not be applicable in regions with different sociocultural contexts. Though we believed most people who meet our leadership definition would likely speak either English, French, or Portuguese, this is a limiting assumption. Conducting the search in additional languages may have yielded more results and contacts. Furthermore, Artificial Intelligence (AI) tools could potentially assist in overcoming this limitation by incorporating multilingual approaches or translation services. Multilingual stakeholders or professional translators could be involved to validate translations’ accuracy. The study team intentionally offered key informant interviews in Amharic in Ethiopia and Malagasy in Madagascar as case studies for the THRIVE study where we had more staff capacity. Additionally, some countries had more extensive lists of stakeholders on their website compared to others, creating a discrepancy with stakeholder quantity. The same challenge was encountered in identifying email addresses, with more extensive online searching required for some countries. The same individual may have been listed more than once due to holding multiple roles in various countries or contexts, thus leading to them being identified multiple times by different members of our team. To address this, during data analysis we removed 334 duplicates leaving us with a list of unique contacts (*n* = 3,545) and an accurate count of the sample’s gender breakdown. Before de-duplicating, we retained stakeholders’ multiple country or organizational affiliations. Deduplication was done in Excel with the Conditional Formatting function, highlighting repeat names and email addresses for the research team to review.

Some governments did not have active or updated websites; thus, we used their social media pages—primarily Facebook and X—to source contact information and relevant in-country organizations, partners, and donors. Social media pages, though useful in identifying relevant names or departments, did not often list contact information. Reliance on social media, including LinkedIn, risks overlooking key stakeholders who may be socio-culturally less likely to be active on social media. These biases may limit the study’s applicability of its recommendations. Lastly, contact information for stakeholders on government and multilateral organizations’ websites was not always updated; we obtained the most accurate contact information from countries where Women in Global Health chapters, GFF contacts, and professional contacts were present and supporting the stakeholder mapping process.

## Implications

This study addresses gender disparities in health leadership by developing a structured and replicable approach to stakeholder mapping for women leaders. The findings of the THRIVE study can be used to influence policy and program development and, more specifically, to design leadership training and capacity-building programs targeting access points, strengths, and facilitators of women’s leadership in RMNCAH-N and immunization in SSA. Findings can be used to advocate for the inclusion of women in decision-making bodies and influence funding priorities by highlighting the impact of women’s contributions in global health. Ministries of Health and NGOs can use this methodology to systematically map, monitor, and analyze poorly-defined populations, enabling data-driven planning and targeted initiatives.

The developed stakeholder mapping methodology has implications that extend well beyond RMNCAH-N and immunization. Its application across SSA demonstrates its scalability and adaptability for other poorly defined populations in diverse regions and settings, such as in low-resource settings where leadership roles may be less formally documented or defined. This methodology can be scaled for broader applications or adapted for stakeholder mapping in other global health areas or regions outside of SSA by taking into account regional, cultural, and contextual differences. Tailoring the sampling universe and source population definitions, engagement strategies, and data sources ensures adaptability to diverse contexts while retaining the methodology’s core principles.

This methodology can be refined by dedicating human resources towards enhancing in-country validation, to better leverage networks and take advantage of the expertise that comes with having eyes on the ground. Partnering with local organizations could enable more thorough cross-checking, snowball sampling, and contextual refinement. For example, increased engagement with local stakeholders in the early stages of developing the framework would produce strategies to overcome potential barriers and to ensure the sample population definition is representative of who is culturally considered to be a leader in RMNCAH-N and immunization. Human resources and financial support for translation services, travel, and in-country validation processes could go a long way in addressing potential sampling biases due to language or technological barriers.

In order to replicate the methodology effectively, team training and cohesion on stakeholder identification criteria, including defining the sampling universe, is necessary to ensure accuracy and completeness of sample. Access and a strong orientation to data collation tools among the team, such as shared spreadsheets and the protocol for screening websites effectively, is necessary for monitoring data and avoiding biases. Multilingual teams with geographic expertise and in-country contacts were essential in providing access to key stakeholders who would have otherwise been unable to be identified online.

Certain ethical implications of data collection and stakeholder engagement must be considered to mitigate negative consequences. Starting from defining the target sample population: it is the responsibility of the study team to carefully consider who represents the population, how the population is defined, how credible population representatives are, and how they are aligned with other community groups or organizations [21]. Bias in data collection activities affects the representativeness and applicability of findings. Misuse of data– such as using it for discriminatory, exploitative, or manipulative purposes– can directly or indirectly harm the sample population. Research teams have an ethical responsibility, and in many cases legal, to ensure that the rights and privacy of individuals and organizations identified during mapping are protected. Privacy protection includes ensuring data, such as stakeholders’ personal and professional information, is kept confidential. When engaging with stakeholders, clear and transparent communication about the study activities, how their data was sourced as well as how it is used and protected is fundamental to facilitating their informed consent. Additional ethical considerations include allocating sufficient time for participants to participate in the study or mapping activity, allowing them to decline or leave the study at any time, and valuing and acknowledging stakeholders for their engagement [22]. The ethical use of advanced tools such as AI, machine learning, or Big Data analytics have the potential to play a role in enhancing stakeholder mapping. Tools’ capacity to analyze large datasets allows research teams to understand key stakeholder trends and gaps and quickly create data visualizations. By enhancing or even automating stakeholder mapping, such as by data mining across multiple languages and platforms, advanced technological tools not only have the potential to increase data quality and robustness, but also enable study teams to divert their time and attention to a critical part of stakeholder engagement: human interaction.

## Conclusion

This paper provides a comprehensive methodology for describing, enumerating, and sampling from stakeholders of a complex, understudied population. This paper’s focus on creating a sampling universe of women leaders in the RMNCAH-N and immunization spaces, specifically within the SSA region, fills a gap in current operational and implementation research, as a dearth of methods exist for systematically sampling a source population across diverse geographies and stakeholder groups across organizational and operational levels.

## Data Availability

No datasets were generated or analysed during the current study.

## Abbreviations

EPI: Expanded Programs on Immunization
FP: Focal point
GFF: Global Financing Facility for Women, Children and Adolescents
ICCs: Inter-agency coordination committees
LO: Liaison officer
LMICs: Low-and middle-income countries
NGO: Non-governmental organization
NITAGs: National Immunization Technical Advisory Groups
OB-GYN: Obstetrics and gynecology
RMNCAH-N: Reproductive, maternal, newborn, child, and adolescent health, and nutrition
SSA: sub-Saharan Africa
UN: United Nations
